# Minimally Invasive Parathyroidectomy

**DOI:** 10.1155/2011/206502

**Published:** 2011-05-23

**Authors:** Lee F. Starker, Annabelle L. Fonseca, Tobias Carling, Robert Udelsman

**Affiliations:** Department of Surgery, Yale University School of Medicine, P.O. Box 208062, New Haven, CT 06520-8062, USA

## Abstract

Minimally invasive parathyroidectomy (MIP) is an operative approach for the treatment of primary hyperparathyroidism (pHPT). Currently, routine use of improved preoperative localization studies, cervical block anesthesia in the conscious patient, and intraoperative parathyroid hormone analyses aid in guiding surgical therapy. MIP requires less surgical dissection causing decreased trauma to tissues, can be performed safely in the ambulatory setting, and is at least as effective as standard cervical exploration. This paper reviews advances in preoperative localization, anesthetic techniques, and intraoperative management of patients undergoing MIP for the treatment of pHPT.

## 1. Introduction

Primary hyperparathyroidism (pHPT) is a common endocrine disease caused by a single parathyroid adenoma in 85% of patients. Identification and resection of the adenoma leads to cure of the disease. Focused unilateral exploration, that is, minimally invasive parathyroidectomy (MIP) of enlarged hyperfunctioning gland(s) under regional anesthetic techniques, has been advocated for the past three decades [1]. Minimally invasive surgery has been defined as the ability of the surgeon to preform traditional surgical procedures employing novel techniques to minimize surgical trauma [2]. In recent years, a number of new minimally invasive techniques have been developed including open minimally invasive parathyroidectomy (MIP) which we describe in this paper. Other minimally invasive approaches, which we will not describe, include video-guided, radio-guided, and endoscopic parathyroidectomies. Prior to current preoperative localization studies, unilateral surgery for pHPT was preformed based upon results of palpation, esophageal imaging, venography, or arteriography [3, 4]. Unilateral focused cervical exploration was also advocated to reduce the cost and morbidity of surgery while maintaining cure rates [5]. Due to limited exposure obtained during MIP, operative adjuncts are required to confirm the adequacy of resection. The intraoperative parathyroid hormone (IOPTH) assay is now recognized by some experts as the single most useful adjunct in this setting [6]. Current imaging technology has enabled preoperative localization of the hyperfunctioning gland, thus allowing an increasing number of focused unilateral exploration. Sestamibi with single-photon emission computed tomography (SPECT), ultrasonography, and more recently high-quality four-dimensional CT (4DCT) scans identify hyperfunctioning glands in the majority of patients. MIP is now routinely performed at high-volume centers with excellent cure rates and minimal morbidity.

A large number of studies have been published over the last decade describing various techniques in minimally invasive parathyroidectomies, and comparing outcomes to each other and to that of conventional bilateral exploration.  Table 1 summarizes these studies. 

The indications for MIP are identical to those for traditional bilateral neck exploration, including patients with symptomatic disease and those with asymptomatic pHPT in accordance with recently published guidelines [7]. In addition to those patients with overt symptoms and signs, there is a large number of patients with significant neurocognitive derangements with biochemically proven pHPT that could potentially benefit from resection [8–10]. 

The current role of MIP in patients with documented or suspected familial pHPT is controversial. In the majority of these patients, conventional bilateral exploration is recommended due to the frequency of multiglandular disease [11].

 In the rare cases (<1%) of suspicion of parathyroid carcinoma as the etiology of pHPT, a radical resection (i.e., an en bloc resection of the involved hyperfunctioning gland with concomitant ipsilateral thyroid lobectomy) should be undertaken during the initial procedure [12]. 

## 2. Preoperative Imaging

The development and refinement of parathyroid imaging has been essential for the development of MIP in the treatment of pHPT. Several noninvasive preoperative localization methods are available, including sestamibi-technetium 99^m^ scintigraphy, ultrasonography, computed tomography (CT), and magnetic resonance imaging (MRI). The most commonly used modality is sestamibi with SPECT, which generates three-dimensional images [12–15]. Technetium 99^m^ methoxyisobutylisonitrile (sestamibi) was initially introduced for cardiac scintigraphy and was incidentally found to be concentrated in parathyroid adenomas [16]. Sestamibi is a monovalent lipophilic cation that diffuses passively through cell membranes and accumulates almost exclusively in mitochondria following negative membrane potentials [17]. Therefore, high mitochondrial density is associated with sestamibi uptake by human parathyroid tissue [18, 19]. The sensitivity of sestamibi scans in the literature is 80% [20, 21]. A major limitation of sestamibi scans is due to the coexistence of thyroid nodules, especially Hürthle cell neoplasms or other metabolically active tissues (e.g., lymph nodes and metastatic tumors) that can mimic parathyroid adenomas thereby causing false-positive results. These concomitant abnormalities have been reported to be as high as 40–48% [22]. A few studies which have evaluated this phenomena saw significant drops in sensitivity of sestamibi scintigraphy when utilized in the presence of a nodular thyroid gland, 96% versus 81% [23] and 92% versus 53% [24]. 

Additionally, the parathyroid tumor cell content may be related to sestamibi uptake. Parathyroid oxyphil cell content >20% quadruples the rate of obtaining a positive sestamibi scan, while small adenomas (<600 mg) with low (<20%) numbers of oxyphil cells are usually associated with a negative scan [25]. Additionally, sestamibi with SPECT does not provide detailed anatomical depiction and can only rarely detect double adenomas and multiglandular hyperplasia [26].

Ultrasound (US) is effective, noninvasive, and inexpensive, but its limitations include both operator dependency and being limited to application in the neck because it cannot image mediastinal tumors. The normal parathyroid gland is generally too small to be visualized sonographically, whereas the parathyroid enlargement seen in pHPT is often identified as a homogeneously hypoechoic extrathyroidal ovoid mass. The accuracy of lateralization and correct quadrant localization of parathyroid tumors is highly variable, especially for ultrasound, due to operatordependence. Similar to sestamibi with SPECT, the ability of an ultrasound to detect the parathyroid abnormality is reduced in milder forms of pHPT (i.e., only mildly elevated PTH and calcium levels) and in obese patients [27]. Thus, some surgeons suggest reserving sestamibi with SPECT for patients with negative US or as an adjunct to patients with concomitant thyroid disease [28, 29]. Additionally, some studies suggest improved sensitivity of parathyroid tumor localization using sestamibi with SPECT and surgeon-performed US [30]. 

**Figure 1:**
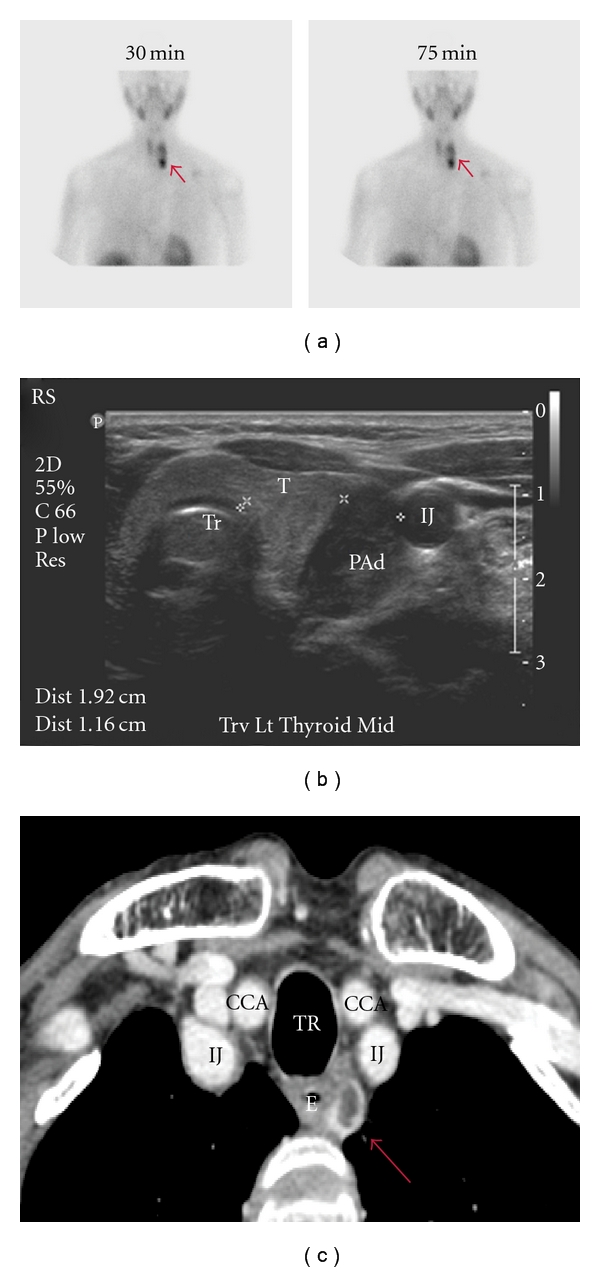
Preoperative imaging in primary hyperparathyroidism. (a) Sestamibi with SPECT displaying focal retention in the left lower position (arrow) after 30 and 75 minutes washout, respectively. (b) Corresponding cervical ultrasound from the same patient showing a large hypoechoic cystic parathyroid adenoma (arrow). The left thyroid lobe (T), trachea (Tr), and left internal jugular vein (IJ) are indicated. (c) Four-dimensional parathyroid CT scan depicting an ectopically positioned left superior paraesophageal parathyroid adenoma (arrow). IJ: internal jugular vein; T: trachea; E: esophagus; CCA: common carotid artery.

Four-dimensional CT scan (4DCT) is a promising new parathyroid imaging technique. 4DCT scan is similar to CT angiography. The term is derived from three-dimensional CT scanning with the additional dimension referring to the changes in perfusion of contrast over time. Exquisitely detailed multiplanar images are obtained to accentuate the differences in the perfusion characteristics of hyperfunctioning parathyroid glands (i.e., rapid uptake and washout), compared with normal parathyroid glands and other structures in the neck (Figure 1). The images provide both anatomic and functional information (based on changes in perfusion) in a single study that the operating surgeon can easily interpret. To date, parathyroid 4DCT has mainly been used as an adjunct to other imaging modalities in the remedial setting [31]. In a study by Rodgers et al., 4DCT displayed improved sensitivity (88%) over sestamibi imaging (65%) and ultrasonography (57%), when these imaging studies were used to lateralize hyperfunctioning parathyroid glands to one side of the neck. Moreover, when used to localize parathyroid tumors to the correct quadrant of the neck (i.e., right inferior, right superior, left inferior, or left superior), the sensitivity of 4D-CT (70%) was significantly higher than sestamibi imaging (33%) and ultrasonography (29%) [31].

Even in patients with negative, discordant, or unconvincing preoperative imaging studies, the cure rates are excellent in experienced hands; however, invasive localization procedures such as selective parathyroid venous sampling (SVS) with measurements of PTH may be indicated. Rapid PTH measurements have been successfully employed in the angiography suite, and interventional radiologists can obtain additional samples from a region in which a subtle but potentially significant PTH gradient is detected [32]. Ultrasonography can be employed to guide preoperative aspiration of a putative parathyroid gland in which PTH is measured to confirm that the suspected tissue is parathyroid. This technique, like venous localization, should be reserved for remedial cases [33]. 

## 3. Anesthesia

**Figure 2:**
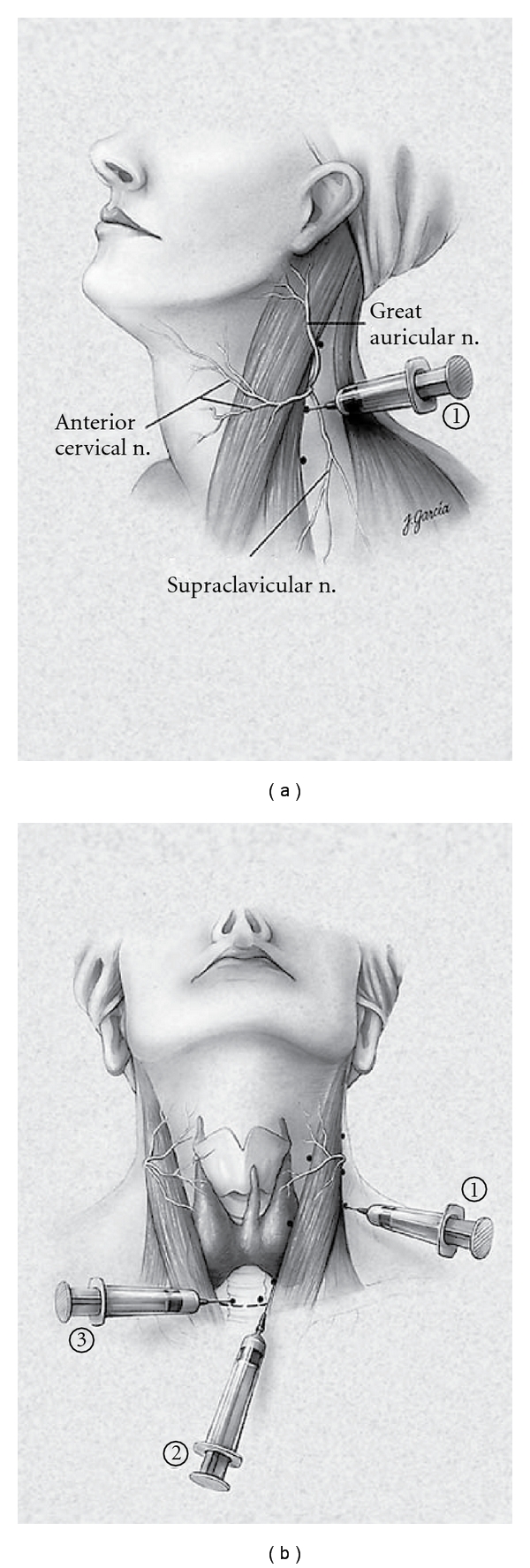
Cervical block anesthesia. (a) A superficial cervical block is administered posterior and deep to the sternocleidomastoid muscle (SCM; 1). (b) Local infiltration is also performed along the anterior border of the SCM [2], followed by a local field block [3]. (From [34], with permission, Copyright 2002, Lippincott Williams &Wilkins).

The majority of parathyroid explorations are performed under general anesthesia utilizing either a standard general endotracheal tube (ETT) or a laryngeal mask airway (LAM). Conversely, high-volume centers have utilized monitored anesthesia care (MAC) with local and regional anesthesia. The regional block can be performed by the surgeon, just prior to incision after mild intravenous sedation has been administered (Figure 2). We use 1% lidocaine containing 1 : 100000 epinephrine employing a total of 20 mL. Intravascular infiltration of local analgesic agents must be avoided. Unilateral infiltration posteriorly at Erb's point (located on the posterior border of the sternocleidomastoid muscle midway between its attachments to the mastoid process, the sternum, and the clavicle) and along the anterior border of the sternocleidomastoid muscle on the side of the localized gland and along the area of incision provides excellent analgesia during virtually every case. Intravenous sedation is maintained to alleviate patient anxiety while maintaining a patient who is capable of phonating. 

Regional anesthesia for the resection of hyperfunctioning parathyroid glands avoids potential complications associated with general anesthesia, which includes endotracheal intubation, which has been associated with up to a 5% risk of vocal cord changes and damage [35]. Furthermore, with patients remaining conscious during exploration, the operating surgeon may assess functional status of phonation. Conversion to general anesthesia is occasionally performed and may be due to concomitant thyroid pathology, concern for parathyroid carcinoma, persistently elevated intraoperative PTH levels, difficulty in ensuring the safety of the recurrent laryngeal nerve, and patient discomfort [3]. Regardless of the reason for conversion in all cases, conversion should be done in a controlled fashion with protection of the surgical field [3]. We recently reported a 10.6% conversion to general anesthesia in a study of 441 consecutive patients [25]. 

## 4. Procedure

**Figure 3:**
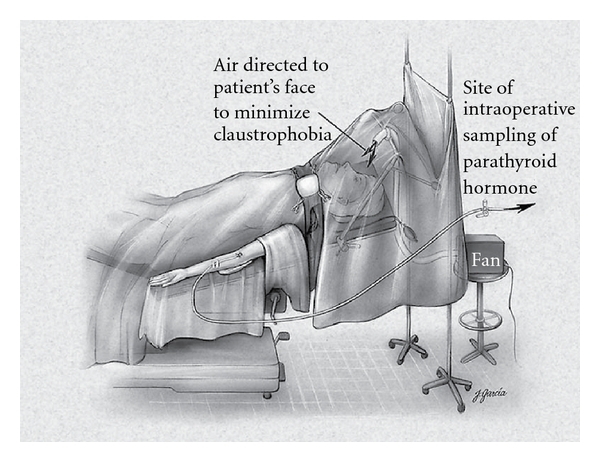
The patient has a large-bore peripheral intravenous line inserted, which is used for medication and fluid administration, as well as sampling for parathyroid hormone (PTH) levels. The patient is awake, and a fan is used to blow room air gently toward his or her face to minimize the sensation of claustrophobia. (From [36], with permission, Copyright 2002, Lippincott Williams & Wilkins).

A 20-gauge intravenous catheter is placed in the antecubital fossa, and a single baseline PTH level is obtained in the preoperative holding area. The patient is transported to the operating room and placed in the semifowler position, and a screen is used to protect the patient's face from the operative field. The anesthesia personal will require access to the intravenous line to obtain PTH measurements (Figure 3) [34]. Moderate sedation is administered, and a regional cervical nerve block is then administered as described. An abbreviated Kocher incision (approximately 3 cm) is then made, and the anterior compartment of the neck entered the median raphe is mobilized, the parathyroid adenoma visualized, the recurrent laryngeal nerve is protected, and the adenoma is excised. 

Once resection is complete, serial blood draws are obtained. Adequacy of resection is confirmed when the patient's preoperative baseline PTH level decreases by at least 50% and returns to the normal range [36–40]. 

## 5. Intraoperative Parathyroid Hormone Monitoring

In 1990, intraoperative PTH monitoring was introduced and allowed for a biochemical alternative to direct four-gland visualization [41]. Prior to this, multiglandular disease could not be accurately ruled out without directly visualizing at least all four glands. Intraoperative PTH monitoring is feasible because PTH has a short half-life of the hormone (3–5 minutes). In some patients, the PTH level fails to decline after resection, thus indicating residual abnormal parathyroid tissue, and further exploration is required. Since the most advantageous time to cure pHPT is during the first surgical exploration, it is the obligation of the surgeon to perform a meticulous exploration, evaluating both eutopic and ectopic sites. This exploration may include the retroesophageal space, thymus gland, carotid sheaths, and submandibular region for undescended glands. If the occult gland is still not identified, additional intraoperative adjuncts may be used, including ultrasound and bilateral internal jugular vein sampling to determine if an ipsilateral PTH gradient is present. This technique has guided us to explore upstream and locate occult undescended or partially descended glands. Partial or complete thyroid lobectomy for intrathyroidal parathyroid tumors can be performed depending on the suspected location of the missing gland. In the case of difficult localization of the hyperfunctioning gland, a traditional bilateral exploration may need to be performed. In the rare case where a mediastinal gland is suspected after bilateral exploration has not revealed the hyperfunctioning gland, a partial sternotomy may rarely be required during initial exploration [42]. 

## 6. Complications

The complications that may be associated with MIP are the same as complications that may be associated with conventional bilateral exploration, and the complication rates are at least as low as those associated with the latter. However, due to the use of regional anesthesia, MIP avoids potential complications associated with general anesthesia, which includes endotracheal intubation, which has been associated with the risk of vocal cord changes and damage. Hematomas and ipsilateral recurrent laryngeal nerve injury may occur with MIP. Temporary hypocalcemia (due to hungry bone syndrome) may also occur. In a prospectively collected and retrospectively reviewed series of 656 consecutive parathyroidectomies performed between 1990 and 2001 (of which 401 were performed in the conventional fashion, and 255 were performed as MIP), we reported no significant difference in complication rates (3% and 1.2%, resp.) or cure rates (97% and 99%, resp.) [43]. 

## 7. Patients with Recurrent or Persistent Disease

Despite modern preoperative imaging techniques, endocrine surgeons are still faced with three distinct groups of patients who present unique and challenging management issues: (1) those with persistent pHPT, (2) or recurrent, and lastly (3) those who have had prior neck surgery, particularly thyroid resections, and develop pHPT. Other situations of difficult neck dissections arise in the setting of parathyroid carcinoma and parathyromatosis due either to carcinoma or spillage during a previous adenoma resection and those patients who have had radiation to the neck and/or mediastinum. Regardless of the pathophysiology, the challenge is the same as remedial surgery in previously violated surgical planes. Reoperative cervical and mediastinal exploration is associated with an overall increase of failure and complication rates [44, 45]. 

## 8. Conclusions

Due to the significant advancements in technology, MIP has become the procedure of choice for sporadic pHPT at specialized centers. Minimally invasive parathyroidectomy has improved cosmetic results due to smaller incisions, decreased surgical trauma leading to less postoperative pain, shorter operative times, and a decreased overall hospital stay. The rate of cure was recently shown to be comparable or improved compared to traditional bilateral neck explorations and can be performed in the outpatient setting. It is to be emphasized that MIP and conventional cervical exploration are both highly successful and associated with low complication rates. Surgeon experience, regardless of the technique employed, is paramount to obtain excellent results. 

## Figures and Tables

**Table 1:** Summary of studies describing technique and outcomes of minimally invasive parathyroidectomy.

Study	Methods	Study type	Success rate	Pre-op	Intra-op
Adil et al. [46]	305 patients (1997–2007)	Retrospective case series	100% success	Pre-op sestamibi	IOPTH gamma probe
Krausz et al. [47]	541 patients	Prospective cohort study	97% success	Pre-op sestamibi scan or ultrasound	IOPTH
Sevinç et al. [48]	56 patients (25 MIP, 31 MIRP)	Prospective study	Similar outcomes	Pre-op sestamibi	IOPTH in MIP gamma probe in MIRP
Sugino et al. [49]	167 patients (80 IOPTH monitoring, 87 without IOPTH monitoring)	Prospective study	97% versus 93%	Pre-op sestamibi scan, ultrasound, or CT scan	
Fouquet et al. [50]	200 patients	Prospective study	72% success 28% converted to open	Pre-op sestamibi scan or ultrasound	IOPTH
Hessman et al. [51]	143 patients (75 MIP, 68 VAP)	Multicenter prospective randomized control trial	Similar outcomes (conversion rate, pain), VAP longer	Pre-op sestamibi scan	IOPTH
Slepavicius et al. [52]	48 patients (24-focused exploration)	Prospective randomized control trial	Similar outcomes	Pre-op sestamibi scan or ultrasound	IOPTH
Shindo et al. [53]	186 patients	Prospective study (historical cohort)	95% success	Pre-op sestamibi scan or ultrasound	IOPTH
Shindo and Rosenthal [54]	88 patients	Retrospective review	91% success	Pre-op sestamibi scan or ultrasound	IOPTH
Rubello et al. [55]	452 patients 344 MIRP	Prospective study	93% success	Pre-op sestamibi scan or ultrasound	IOPTH gamma probe
Lindekleiv et al. [56]	47 patients	Retrospective study	97% success	Pre-op sestamibi scan	IOPTH
Aarum et al. [57]	50 patients 23 MIP, 26 conventional	Prospective randomized control trial	96% versus 94%	Pre-op sestamibi and ultrasound	IOPTH
Tang et al. [58]	202 patients 50 MIP, 152 open	Retrospective study	Improved quality of life		
Politz et al. [59]	152 patients (118 MIP, 34 open)	Retrospective study	98% success	Pre-op sestamibi scan	Intra-op gamma probe
Pang et al. [60]	500 patients prospective	Prospective study	97.4% success	Pre-op sestamibi scan + ultrasound for incision placement	
Soon et al. [61]	699 patients	Retrospective study			IOPTH (not used as management aid)
Mihai et al. [62]	298 patients 182-sestamibi 150-MIP	Prospective study	97.3% success	Pre-op sestamibi scan	
Caudle et al. [63]	140 patients	Prospective study	96% success	Pre-op sestamibi	Intra-op gamma probe, frozen section
Alfadda et al. [64]	55 patients	Retrospective study	93% success		
Carling et al. [43]	441 patients	Prospective case series	10% conversion to GA (thyroid exploration/conversion to open exploration/patient discomfort)	Pre-op sestamibi scan or ultrasound	IOPTH
Barczynski et al. [65]	60 patients 30 MIP, 30 VAP	Prospective randomized control trial	97% success in both groups, VAP with decreased pain & need for analgesia	Pre-op sestamibi and ultrasound	IOPTH
Cohen et al. [66]	130 patients	Retrospective review	98.6% success	Pre-op sestamibi scan and/or ultrasound	IOPTH
Ollila et al. [67]	77 patients	Retrospective review	96% success	Pre-op sestamibi scan	Intra-op gamma probe, selective frozen section
Miccoli et al. [68]	51 patients 26-RA, 25-GA	Prospective randomized control trial	Decreased operative time and post-op need for analgesia with RA	Pre-op sestamibi scan and ultrasound	IOPTH
Mekel et al. [69]	146 patients 84 MIP (79%)	Prospective study		Pre-op sestamibi scan or ultrasound	IOPTH
Grant et al. [70]	1361 patients	Retrospective review	54% open exploration 44% MIP (2% conversion)		
Rubello et al. [71]	268 patients	Retrospective review	96.8% success	Pre-op sestamibi and/or ultrasound	IOPTH
Bergenfelz et al. [72]	50 patients 25-MIVAP, 25-open	Prospective randomized control trial	Decreased operating time with MIP	Pre-op sestamibi scan	IOPTH
